# Nutrients and perinatal depression: a systematic review

**DOI:** 10.1017/jns.2017.58

**Published:** 2017-12-20

**Authors:** Thalia M. Sparling, Robin C. Nesbitt, Nicholas Henschke, Sabine Gabrysch

**Affiliations:** Institute of Public Health, Heidelberg University, Im Neuenheimer Feld 324, 69120 Heidelberg, Germany

**Keywords:** Nutrients, Nutritional biomarkers, Maternal health, Perinatal depression, CES-D, Center for Epidemiological Studies Depression Scale, EPDS, Edinburgh Postpartum Depression Scale, LMIC, low-income and middle-income countries, RCT, randomised controlled trial

## Abstract

Pregnancy and lactation deplete nutrients essential to the neurotransmission system. This may be one reason for the increased risk of depression during the perinatal period. The objective of the present review was to systematically review the literature and summarise evidence on whether blood nutrient levels influence the risk of perinatal depression. PubMed, EMBASE and CINAHL databases were searched for studies of any design. A total of twenty-four articles of different designs were included, representing 14 262 subjects. We extracted data on study population, depression prevalence, nutrients examined, deficiency prevalence, timing of assessment, reporting, analysis strategy and adjustment factors. In all, fourteen studies found associations of perinatal depression with lower levels of folate, vitamin D, Fe, Se, Zn, and fats and fatty acids, while two studies found associations between perinatal depression and higher nutrient levels, and eight studies found no evidence of an association. Only ten studies had low risk of bias. Given the methodological limitations and heterogeneity of study approaches and results, the evidence for a causal link between nutritional biomarkers and perinatal depression is still inconclusive. High-quality studies in deficient populations are needed.

## Introduction

Perinatal depression, also referred to as maternal depression, can occur during pregnancy or up to 1 year postpartum. It is considered the most common complication of pregnancy. In their systematic review of perinatal depression, Gavin *et al*.^(^^1^^)^ reported a pooled point prevalence of 11 % for minor depression during pregnancy and 13 % for postpartum depression in high-income countries. Early studies reported differing levels of perinatal depression in low-income and middle-income countries (LMIC), but the current literature shows that prevalence is consistently higher than in high-income countries^(^^2^^)^. This is thought to be concomitant with or exacerbated by poverty, gender inequity, anxiety, and familial and political instability^(^^3^^,^^4^^)^. In a systematic review focusing on LMIC, pooled prevalence for depressive symptoms was 16 % in the antenatal period (from thirteen studies), and 20 % in the postpartum period (from thirty-four studies)^(^^5^^)^.

Perinatal depression can have serious and long-term adverse effects on women and their children, and is therefore gaining attention in the international health community^(^^2^^,^^6^^–^^8^^)^. Consequences for women include poor self-care, compromised care-giving and increased morbidity from other causes, while for children, malnutrition, poor physical and cognitive development and increased illness have been reported^(^^9^^–^^12^^)^. Strategies to address perinatal depression are, however, less clear, ranging from pharmacological treatment to community-based support mechanisms and general poverty alleviation, and focus primarily on treatment, rather than on prevention^(^^4^^,^^13^^)^.

Various biologically plausible pathways between nutrient levels and depression have been suggested^(^^14^^)^. Many essential nutrients, or compounds derived from such nutrients, are required for the synthesis of neurotransmitters and their modulation, and may therefore be involved in mood regulation^(^^15^^,^^16^^)^. The biochemical role of nutrients in the nervous system is described in detail elsewhere^(^^14^^,^^15^^,^^17^^)^. Pregnancy and lactation place additional demands on a woman's body, and therefore nutrient deficiencies arise more easily during this time. Deficiency of nutrients during this critical period therefore may contribute to the increased risk of depression during the perinatal period. Hormonal shifts and lifestyle changes may in themselves increase the risk of perinatal depression, but may also contribute to changes in nutrient levels and could thus act through that pathway^(^^18^^)^.

Previous reviews limited the measures of nutritional status to one specific nutrient or class of nutrients, such as PUFA^(^^19^^)^. Some explored depression in general (not limited to the perinatal period), and others were not systematic^(^^17^^,^^20^^)^. One review on nutrient supplementation mixed prevention (in previously undiagnosed women) and treatment (in women already diagnosed with depression)^(^^21^^)^. The present review includes published evidence on any nutrient, but the available studies focus on only a few out of the many important nutrients and phytochemicals that may be associated with perinatal depression.

### Objective

We initially designed the present review to summarise the existing evidence from all analytical studies available on the association between nutritional status and perinatal depression in women. The results of the search highlighted two main ways to capture nutritional status: through blood levels (i.e. biomarkers) and through dietary intake measures, each with their particular strengths. Biomarkers are a precise, direct measure of nutritional status that account for bioavailability, while dietary intake data can easily capture a larger panel of nutrients and study the effect of whole diets, thus considering complex food interactions. Studies measuring dietary intake have been published in a separate review^(^^22^^)^. The present review focuses on nutritional biomarkers and their link to perinatal depression.

## Methods

### Search strategy

**Fig. 1.**
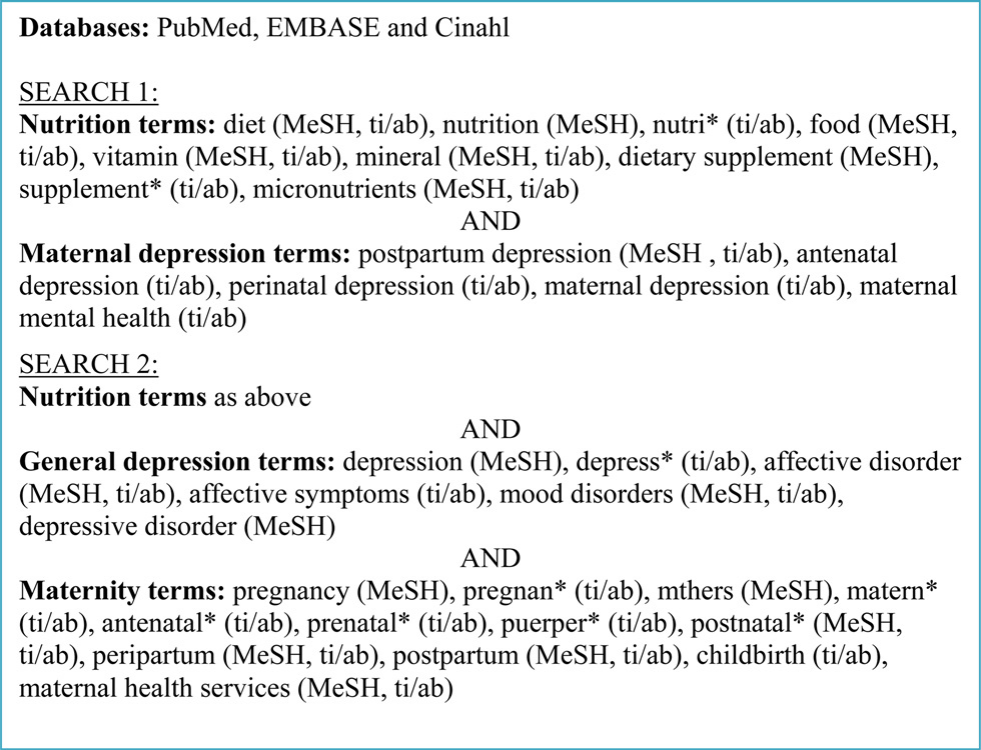
Search strategy. MeSH, medical subject headings; ti/ab, title or abstract.

We searched PubMed, EMBASE and CINAHL databases from 1966 to 1 June 2016 for publications in English. We used both medical subject heading terms and free text keywords in two separate searches to identify all relevant studies. The first search combined nutrition terms with maternal depression terms, while the second combined nutrition terms with depression terms and maternity terms separately (Fig. 1). All titles and abstracts identified by the search were screened by two independent researchers who then reviewed the full text of potentially eligible articles for inclusion.

### Eligibility

We considered articles eligible if they were peer-reviewed and presented measures of association between nutritional biomarkers during or after pregnancy, and depression during pregnancy or up to 1 year postpartum. Analytical studies of any design were eligible for inclusion.

We included studies that evaluated nutritional biomarker levels using blood analysis. We excluded supplementation studies unless blood levels of nutrients were directly compared against perinatal depression. Studies that assessed hormones or other compounds synthesised by the body but not directly affected by dietary intake were not included.

We excluded studies in women who were already diagnosed with depressive symptoms or had other underlying health problems (e.g. HIV). Studies had to assess perinatal depression through a validated depression screening tool (i.e. Edinburgh Postpartum Depression Scale (EPDS); Center for Epidemiologic Studies Depression Scale (CES-D); Beck Depression Inventory; Mini International Neuropsychiatric Interview; twenty-item Self-Reporting Questionnaire; ten-item Kessler Psychological Distress Scale; Goldberg's Depression Scale; Symptom Checklist-90-R, or Kitgum Maternal Mood Scale; Postpartum Depression Screening Scale) or through clinical diagnosis from a trained interviewer; though we also allowed prescription of antidepressants as a proxy for a clinical diagnosis. Two common tools used to screen for depression, the CES-D and the Beck Depression Inventory, have often been used in pregnancy and postpartum as they do not emphasise somatic symptoms such as appetite changes and sleep disturbance. They have been found accurate in screening for both minor and major depression^(^^23^^)^. The only screening tools that totally exclude somatic symptoms and are technically validated for the pregnancy and postpartum period are the EPDS and the Postpartum Depression Screening Scale. These have higher sensitivity than other tools^(^^24^^,^^25^^)^ and we therefore assigned a lower risk of bias to studies using these tools.

### Risk of bias assessment

In order to assess the methodological quality of each study, the Cochrane Collaboration tool^(^^26^^)^ was used to evaluate the risk of bias in the randomised controlled trials (RCT), while observational studies were evaluated using a modified version of the Quality in Prognostic Studies (QUIPS) tool^(^^27^^)^. Two reviewers independently ranked each study as having high, medium/unclear or low risk of bias in each domain of the respective tools. The reviewers compared scores, disagreements were discussed and a consensus reached.

The Cochrane tool comprises the following six domains: random sequence generation (selection bias), allocation concealment (selection bias), blinding of participants and personnel (performance bias), blinding of outcome assessment (detection bias), incomplete outcome data (attrition bias), and selective reporting (reporting bias).

While the QUIPS tool is designed to evaluate bias in prognostic studies, ‘risks’ in epidemiology are similar to ‘prognoses’. It comprises six domains: study participation, study attrition (replaced by response in cross-sectional studies and by selection of controls in the two case–control studies), prognostic factor (exposure) measurement, outcome measurement, confounding, and statistical analysis and reporting. Further details are provided in Supplementary Appendix S1.

### Data extraction and synthesis

We used a standard form to extract characteristics and overall results from all included studies (Table 1). We also extracted information on the type of analysis performed and detailed results for all studies (Supplementary Appendix 2, Tables S1–S4). Studies that examined both B vitamins and Fe biomarkers are described in the two respective categories in the text, but presented as their own group in the tables in order to avoid duplication. Studies examined many different nutrients and their timing of exposure and outcome assessment varied widely. More importantly, several studies only reported linear depression outcomes (as opposed to the appropriate categorical outcome for screening tools), which precluded performing a meta-analysis of associations between nutritional biomarkers and perinatal depression without access to the original data. We therefore present a descriptive summary of the results and methodological quality of all included studies.

**Table 1.** Summary of studies evaluating associations between nutrients and perinatal depression*†‡§||

Study characteristics						Exposure			Outcome		Results	
First author (year)	Country (cohort)	*n*	Study design	Depression prevalence	Nutritional deficiency	Nutrients analysed	Exposure definition	Time assessed	Tool; cut-point	Time assessed	Adj. model	Protective associations
B vitamins												
Blunden (2012)^(^^41^^)^	UK (Southampton Women's Survey)	2856	Cohort	32 %	0·3 %	Folate	Mean nutrient levels	1st trimester	EPDS; 13	PP: 6 months, 1 year	No	None
Chong (2014)^(^^34^^)^	Singapore	709	Cohort	7 % preg, 10 % PP	Not reported	Folate and vitamin B_12_	Mean nutrient levels, quartiles of nutrient levels	3rd trimester	EPDS; 15 preg, 13 PP	3rd trimester, PP: 3 months	Yes	High *v*. low folate levels; only in antenatal period
Vitamin D												
Accortt (2016)^(^^44^^)^	USA (DOMInO trial)	91	Cohort	12 %	85 % ≤20 ng/ml	Vitamin D	Linear nutrient levels	1st trimester	EPDS, continuous	PP: 4–6 weeks	Yes	None (high *v*. low levels of vitamin D as linear trend borderline significant)
Brandenbarg (2012)^(^^46^^)^	Netherlands (Amsterdam Born Children and Their Development (ABCD) study)	4101	Cohort	28 %	44 % ≤20 ng/ml	Vitamin D	Deficient, insufficient, sufficient, and normal	1st trimester	CES-D; 16	2nd trimester	Yes	Normal and sufficient *v*. low levels of vitamin D, linear trend
Cassidy-Bushrow (2012)^(^^33^^)^	USA (DOMInO trial)	178	Cohort	42 %	83 % ≤20 ng/ml	Vitamin D	Linear nutrient levels	1st trimester	CES-D; 16	2nd trimester	Yes	High *v*. low levels of vitamin D as linear trend
Fu (2015)^(^^49^^)^	China	213	Cohort	12 %	83 % ≤20 ng/ml	Vitamin D	Deficient, insufficient and normal	PP: 48 h	EPDS; 12	PP: 3 months	Yes	High *v*. low levels of vitamin D
Gould (2015)^(^^45^^)^	Australia	1040	Cohort (in PUFA RCT)	10 %	42 % ≤20 ng/ml	Vitamin D	Three nutrient levels	PP: 48 h	EPDS; 12	PP: 6 weeks, 6 months	Yes	None (high *v*. low levels of vitamin D only in control group at 6 weeks)
Gur (2014)^(^^48^^)^	Turkey	189	Cohort	24 % at 6 months	40 % ≤20 ng/ml	Vitamin D	Normal, mild deficiency and severe deficiency; means; linear	2nd trimester	EPDS; 12	PP: 1 week, 6 weeks, 6 months	No	High *v*. low levels of vitamin D
Murphy (2010)^(^^50^^)^	USA	97	Cohort	12 %	58 % ≤32 ng/ml	Vitamin D	Insufficient *v*. sufficient	PP: each month for 1–7 months	EPDS; 9	PP: each month for 1–7 months	Yes	Sufficient *v*. insufficient levels of vitamin D
Robinson (2014)^(^^43^^)^	Australia (Raine: Western Australian Pregnancy Cohort Study)	796	Cohort	19 %	24 % <19 ng/ml	Vitamin D	Quartiles of nutrient levels	2nd trimester	Shortened EPDS; 6	PP: 3 d	Yes	High *v*. low levels of vitamin D
Huang (2014)^(^^42^^)^	USA	498	Cross-section	12 %	4 % ≤20 ng/ml	Vitamin D	Quartiles and linear trend of nutrient levels	2nd trimester	DASS-21, PHQ-9; linear	2nd trimester	Yes	None
Nielsen (2013)^(^^38^^)^	Denmark (Danish National Birth Cohort)	1480	Case–control	N/A	Not reported	Vitamin D	Six nutrient levels	3rd trimester	Prescription filled without admission	Within 1 year	Yes	Normal *v*. high levels of vitamin D
Fe and B vitamins												
Lukose (2014)^(^^29^^)^	India	365	Cross-section (baseline of RCT)	33 %	30 % anaemic	Hb, complete blood count, erythrocyte DW, vitamin B_12_, Hcy, MMA, folate	Low *v*. normal nutrient levels	1st trimester	K-10; 6	1st trimester	Yes	Anaemia *v*. no anaemia
Watanabe (2010)^(^^32^^)^	Japan	86	Cross-section	62 %	Not reported	Folate, Hcy, total protein, albumin, Fe, Hb, Hct	Mean nutrient levels, low *v*. high concentrations of folate and Hcy	1st trimester	CES-D; 16	1st trimester	Yes	None
Fe												
Albacar (2011)^(^^36^^)^	Spain	729	Cohort	9 % major depression	Fe depletion: 25 %, Fe deficiency: 14 %	Fer, transferrin, free TfS, CRP	Fe depletion, marginal Fe depletion, Fe deficiency	PP: 48 h	SCID-CV	48 h, 8 weeks, 32 weeks	Yes	Ferritin levels above Fe deficiency and Fe depletion *v*. below
Aubuchon-Endsley (2012)^(^^28^^)^	USA	82	Cross-section	Not reported	No deficiency	Hb, sTfR, Fer, AGP	Mean nutrient levels	PP: 3 months	SCL-90-R; mean and sd	PP: 3 months	No	None
Bae (2010)^(^^31^^)^	Korea	114	Cross-section	43 %	No anaemia	Leucocytes, erythrocytes, Hb, Hct, MCV, MCH, MCHC, erythrocyte DW, platelets, PDW, MPV	Mean nutrient levels. No cut-offs	Pregnancy	BDI; 10	Pregnancy	No	None
Se												
Mokhber (2011)^(^^37^^)^	Iran	166	RCT	Not reported	Not reported	Se *v*. placebo	Randomised; supplementation during pregnancy	N/A	EPDS; mean difference	PP: 0–8 weeks	N/A	Se supplementation *v*. no supplementation
Zn and Mg												
Wójcik (2006)^(^^47^^)^	Poland	66	Cohort (all supplement)	3 d PP: 42 %, 30 d PP: 29 %	Not reported	Zn and Mg	Mean nutrient levels	PP: 3 d, 30 d	EPDS; 10	PP: 3 d, 30 d	No	High *v*. low levels of Zn
Fats and fatty acids												
Markhus (2013)^(^^35^^)^	Norway	43	Cohort	7 %	28 % ≤5·1 on omega-3 index	DPA, DHA, omega-3 index, *n*-6:*n*-3 ratio, total HUFA score, and *n*-3 HUFA score	Mean nutrient levels and quartiles of nutrients	3rd trimester	EPDS; 10 or continuous	PP: 3 months, 6 months, 12 months	No	High *v*. low omega-3 index scores
Pinto (2017)^(^^51^^)^	Brazil (Mental Health and Nutritional Status During Pregnancy and Postpartum)	172	Cohort	23 % (average of three trimesters)	Not reported	ALA, EPA, DPA, DHA, LA, AA, γ-LA, EDA, ETE, total *n*-6:*n*-3 ratio	Mean nutrient levels. No cut-offs	1st, 2nd and 3rd trimesters	EPDS; 11	1st, 2nd and 3rd trimesters	Yes	High *v*. low *n*-3 PUFA levels; low *v*. high total *n*-6:*n*-3 ratio
Teofilo (2014)^(^^52^^)^	Brazil (Mental Health and Nutritional Status During Pregnancy and Postpartum)	238	Cohort	22 % (2nd and 3rd trimesters)	Not reported	TAG, total cholesterol, LDL, HDL	Mean nutrient levels. No cut-offs	1st, 2nd and 3rd trimesters	EPDS; 11	1st, 2nd and 3rd trimesters	Yes	High *v*. low HDL concentration
Rees (2009)^(^^39^^)^	Australia	38	Case–control	N/A	Not reported	*n*-3 PUFA, DHA, EPA, ALA, *n*-6 PUFA, LA, AA, DPA, *n*-6:*n*-3 ratio, AA:EPA and DHA:DPA	High *v*. low nutrient levels	3rd trimester	EPDS; 13, SCID-CV	3rd trimester	Yes	High *v*. low total *n*-3 content, DHA and *n*-6:*n*-3 ratio
All nutrients												
Bodnar (2012)^(^^40^^)^	USA (Antidepressant Use During Pregnancy (ADUP) Study)	135	Cohort	22 %	‘Population was well-nourished’	AA, EPA, DHA, folate, Hcy, vitamin C, vitamin D, vitamin A and carotenoids, vitamin E, Fer and sTfR	Tertiles of factors identified with PCA: EFA, micronutrients, carotenoids	1st or 2nd trimester	SCID-CV	2nd or 3rd trimester	Yes	None

* Statistical terms: Adj. model, statistical analysis adjusted for potential confounding factors; N/A, not applicable; PCA, principal components analysis; RCT, randomised control trial.

† Depression terms: BDI, Beck Depression Inventory; CES-D, Center for Epidemiological Studies Depression Scale; DASS-21, Depression, Anxiety, and Stress Scales; EPDS, Edinburgh Postpartum Depression Scale; K-10, Kessler Depression Scale; PHQ-9, Patient Health Questionnaire Depression Module; PP, postpartum; preg, pregnancy; SCID-CV, Structured Clinical Interview; SCL-90-R, Symptom Checklist-90-Revised.

‡ Fe and blood terms: AGP, inflammatory marker 1-acid glycoprotein; CRP, inflammatory marker C-reactive protein; DW, distribution width; Fer, ferritin; Hct, haematocrit; MCH, mean corpuscular Hb; MCHC, mean corpuscular Hb concentration; MCV, mean corpuscular volume; MPV, mean platelet volume; PDW, platelet distribution width; sTfR, soluble transferrin receptors; TfS, free Fe and transferrin saturation.

§ Vitamins and minerals: A, retinol; B_12_, cobalamin; C, ascorbic acid; D, serum 25-hydroxyvitamin D; EFA, essential fatty acids; Hcy, homocysteine; MMA, methylmalonic acid.

ǁ Fatty acid terms: AA, arachidonic acid; ALA, α-linolenic acid; EDA, eicosadienoic acid; ETE, eicosatrienoic acid; HUFA, highly unsaturated fatty acids; LA, linolenic acid.

## Results

### Study inclusion and characteristics

**Fig. 2.**
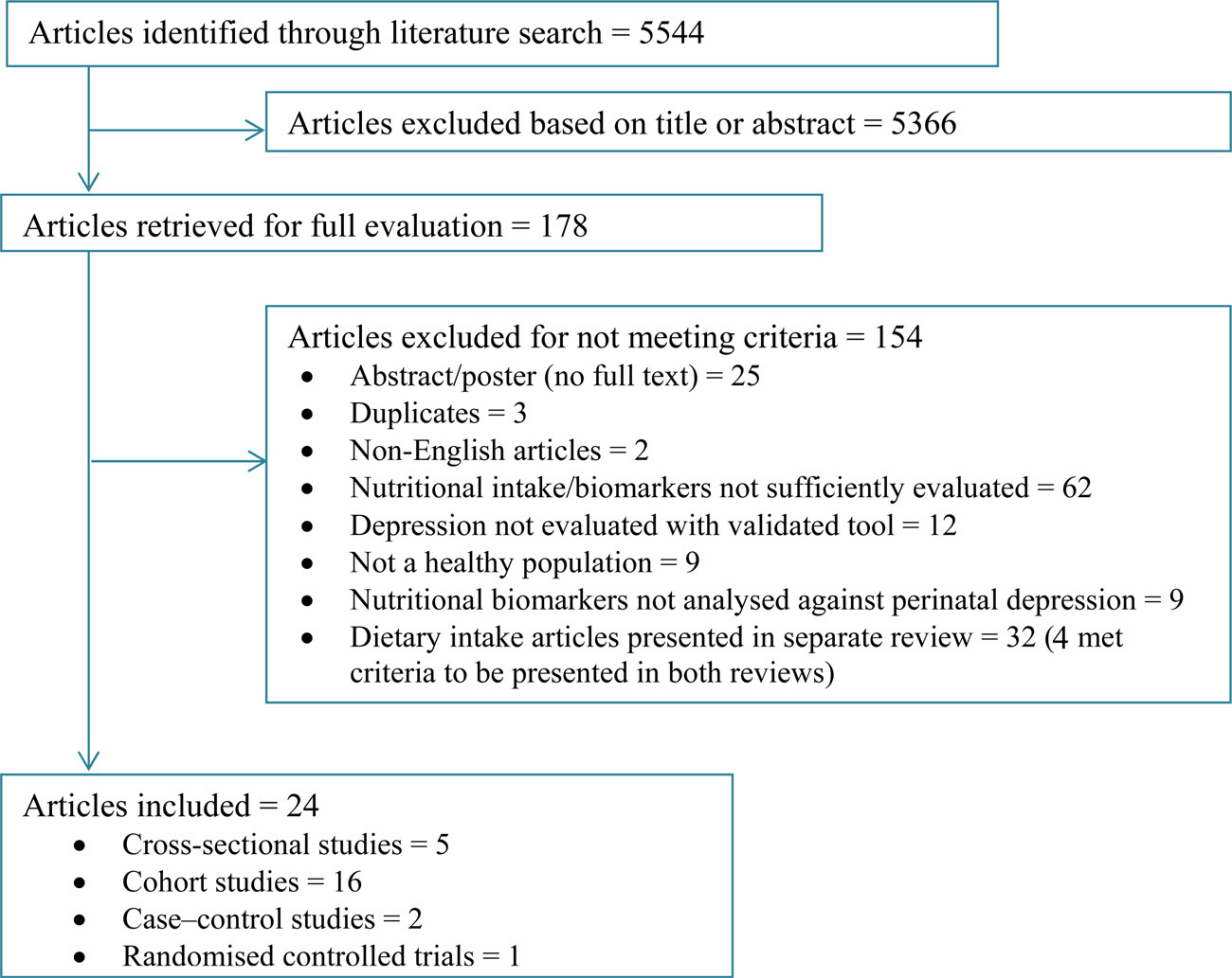
Flowchart for inclusion of studies.

From three databases, 5544 articles were identified after removing duplicates. Titles and abstracts were screened and 178 full-text articles were retrieved and checked against inclusion criteria. A total of thirty-five articles that used diet or intake measures to assess nutritional status were reviewed separately^(^^22^^)^, while four articles fit inclusion criteria and were included in both reviews. We finally included twenty-four articles in this review, representing 14 262 participants from sixteen mainly high-income countries (Fig. 2).

Studies were grouped in four categories according to the nutrients measured: (1) vitamins (four B vitamin studies – two of which also included Fe and are presented in that category as well, and ten vitamin D studies); (2) minerals (five Fe studies, one Se study and one Zn and Mg study); (3) fat and fatty acids (three studies assessing PUFA and one assessing lipoproteins and cholesterol); and (4) several essential nutrients (one study assessing major essential nutrients grouped by principal components analysis). In terms of study population, five studies used population-based prospective cohorts, seventeen recruited participants from hospitals or clinics, one was a population-based convenience sample^(^^28^^)^, and one was a baseline assessment of an RCT^(^^29^^)^.

Of the studies, three used the Structured Clinical Interview for Diagnostic and Statistical Manual of Mental Disorders, 4th edition (DSM-IV) Diagnoses (SCID-CV), and twenty used a screening tool, most commonly the EPDS (fifteen studies) or the CES-D (three studies). One study used filling a prescription for antidepressants as a proxy for clinical diagnosis. Most studies reported a prevalence of perinatal depression between 10 and 30 %, consistent with literature reviews^(^^10^^,^^30^^)^. Five studies found a depression prevalence over 30 % in their populations, of which three over 40 %^(^^31^^–^^33^^)^, while three studies reported prevalences lower than 10 %^(^^34^^–^^36^^)^. Two studies failed to report depression prevalence and another two could not estimate it as they used a case–control design^(^^28^^,^^37^^–^^39^^)^.

Of the twenty-four included studies, sixteen reported the prevalence of nutrient deficiency for their nutrient of interest. Five reported that either none or a very small percentage (<5 %) of their study population was deficient^(^^28^^,^^31^^,^^40^^–^^42^^)^. Interestingly, the eight studies that found no association between nutritional biomarkers and perinatal depression included those five, and one further study that did not report its nutrient deficiency prevalence^(^^32^^)^.

The reported prevalence of nutrient deficiency was highest in the ten vitamin D studies. Using a common cut-off for vitamin D deficiency of 20 ng/ml and converting nmol/l to ng/ml, deficiency in the study populations ranged from 24 %^(^^43^^)^ to 85 %^(^^44^^)^. In five studies examining Fe and Fe-deficiency anaemia, two (one American and one Korean) reported no deficiency or anaemia^(^^28^^,^^31^^)^, while a study from India reported 30 % anaemia prevalence, based on Hb concentrations, without assessing ferritin concentrations or other markers that differentiate Fe-deficiency anaemia from other forms^(^^29^^)^. Another found 14 % Fe deficiency and 25 % Fe depletion in a Spanish population^(^^36^^)^. One Japanese study on Fe did not present the prevalence of deficiency^(^^32^^)^. A Norwegian group suggested that being at <5 % of the omega-3 PUFA index could be indicative of a biological deficiency. Their population was categorised into percentiles, and 28 % (12/43) subjects were at or below 5·1 % of the PUFA index, which they hypothesise indicates deficiency in fatty acids^(^^35^^)^. Eight studies did not report whether there were nutritional deficiencies in their populations.

### Risk of bias assessment

**Fig. 3.**
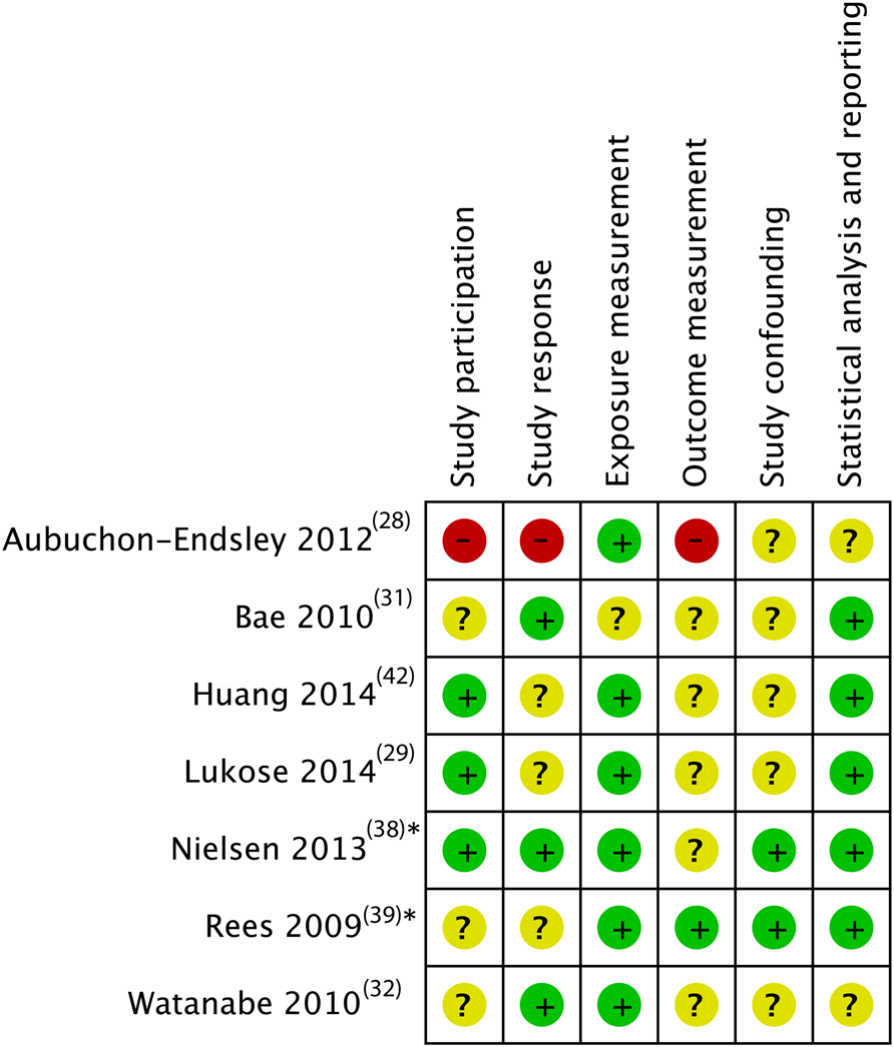
Risk of bias assessment results for cross-sectional studies and case–control studies. * Case–control studies; +, low risk of bias; ?, unclear risk of bias; –, high risk of bias.

**Fig. 4.**
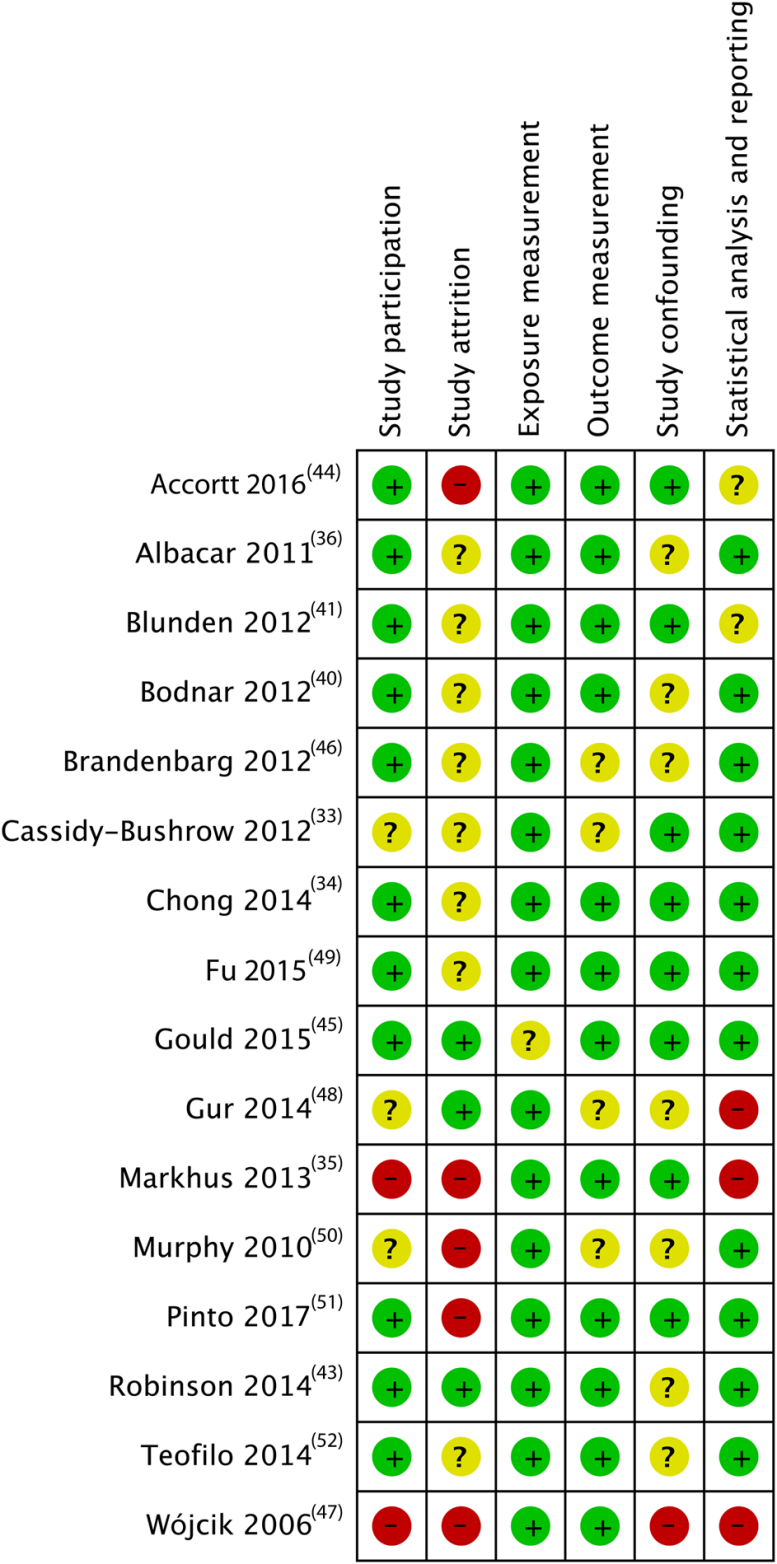
Risk of bias assessment results for cohort studies. +, Low risk of bias; ?, unclear risk of bias; –, high risk of bias.

All studies had potential sources of bias in at least one domain. We present the risk of bias of the five cross-sectional and two case–control studies in Fig. 3, and of the sixteen cohort studies in Fig. 4. Since there was only one RCT, we describe it in the text.

All of the cross-sectional and case–control studies had unclear or medium to high risk of bias in at least two domains, often because they failed to report crucial information. The domains ‘exposure measurement’ and ‘statistical analysis and reporting’ were those with the lowest risk of bias. Outcome measurement and confounding were most problematic, with only one study considered at low risk of bias in each of these domains. Even in adjusted models, four out of seven studies did not include important confounders.

The sixteen cohort studies had low risk of bias in exposure measurement, with one study using umbilical cord blood instead of maternal venous blood^(^^45^^)^. Outcome measurement introduced little bias for this study design; only two studies had medium risk of bias for using the CES-D as a screening tool^(^^33^^,^^46^^)^. Eleven out of sixteen cohort studies had a low risk of bias in study participation, whereas five studies had potential bias in their sampling and selection of participants. Study attrition was the domain with the highest risk of bias, with only three studies having low risk of bias. Three cohort studies lost about half of their study population to follow-up^(^^35^^,^^37^^,^^44^^,^^47^^)^. Of the eleven studies with over 10 % loss to follow-up, six failed to analyse differences between completers and non-completers, which meant they had high risk of bias in this domain. Only eight of sixteen studies adequately adjusted for confounders, while the remainder had a medium or high risk of bias due to their failure to account for variables such as history of depression, social or marital support, or basic demographic factors. Finally, in the domain of statistical analysis and reporting, twelve of the sixteen cohort studies had a low risk of bias as they clearly presented adjusted models with effect estimates, two studies had medium risk of bias for unclear reporting, and two others were considered at high risk of bias for failure to present adjusted models. The RCT by Mokhber *et al*.^(^^37^^)^ had low performance and detection bias, medium risk of selection and reporting bias, and high risk of attrition bias, with only 51 % of participants completing the trial.

### Results from studies

Fourteen studies reported protective effects from higher (*v.* lower) nutrient levels (B vitamins, vitamin D, Fe, Se, Zn and PUFA): one cross-sectional study, one case–control study, eleven cohort studies and one RCT (see Table 1), three of which did not present an adjusted analysis^(^^35^^,^^47^^,^^48^^)^. Two studies found that higher nutrient levels were associated with higher risk of depression^(^^29^^,^^38^^)^. Eight studies, four cohort and four cross-sectional, found no statistically significant association between nutrient levels studied and perinatal depression in their main analysis (Table 1). All studies are described below in four categories, according to the nutrient groups examined.

#### Vitamins (Supplementary Appendix S2, Table S1)

There was little evidence for any association between folate or vitamin B_12_ and perinatal depression from the four included studies. Of the four studies, three had low risk of bias in at least three of the six domains^(^^29^^,^^34^^,^^41^^)^. Chong *et al*.^(^^34^^)^ examined folate and vitamin B_12_ in a cohort in Singapore in the third trimester of pregnancy and measured depression at baseline and at 3 months postpartum. Vitamin B_12_ was not associated with depression at either time point. Increased folate levels were protective against depression in the antenatal assessment (OR 0·69 per standard deviation increase (95 % CI 0·52, 0·94); *P* = 0·02). However, at 3 months postpartum when including the antenatal depression score in the model, the relationship was weaker and no longer significant (OR 0·84 (95 % CI 0·62, 1·12); *P* = 0·25). An English cohort study assessed whether folate levels in the first trimester were related to depression at 6 and 12 months postpartum and found no association at either time point^(^^41^^)^. In two cross-sectional studies, one from India and one from Japan, researchers found no association between B vitamins and perinatal depression^(^^29^^,^^32^^)^.

Evidence of a protective effect on perinatal depression was stronger for vitamin D, but not entirely consistent. Out of ten studies on the topic, four did not find a significant protective association in their main analysis (one had high risk of bias in several domains, one had medium risk of bias in several domains and one had low risk of bias in most domains). One study found no association^(^^42^^)^ and a small cohort study with high loss to follow-up found a borderline protective association (log-transformed continuous vitamin D levels, per unit: *β* = −0·21; *P* = 0·06)^(^^44^^)^. A large Australian study, within a PUFA trial, found no association overall, but within the control group at 6 weeks postpartum, vitamin D levels in cord blood were protective against depression (reference: <10 ng/ml; 10·1–20 ng/ml: risk ratio 0·35 (95 % CI 0·17, 0·69); >20 ng/ml: risk ratio 0·24 (95 % CI 0·12, 0·51)), with strong evidence for the interaction with treatment (*P* = 0·006)^(^^45^^)^. A Danish case–control study with low risk of bias reported that higher levels of vitamin D were associated with more depression^(^^38^^)^.

The other six vitamin D studies not only reported protective associations but found linear trends between vitamin D concentrations and depression. A study from the Netherlands reported that for each 10 nm decrease in vitamin D concentration, there was a 5 % increase in risk of depressive symptoms, and for the most deficient category (≤29·9 nmol/l; ≤11·9 ng/ml), the adjusted OR was 1·48 (95 % CI 1·13, 1·95)^(^^46^^)^. This study had moderate risk of bias in three domains and low risk of bias in three domains. A Chinese cohort study with low risk of bias in five of six domains found a protective effect of vitamin D levels on postpartum depression (OR 0·81 (95 % CI 0·70, 0·92); *P* < 0·001)^(^^49^^)^. Two small studies, one Turkish and one American, both with high risk of bias in at least one domain, found higher vitamin D levels to be protective against depression^(^^48^^,^^50^^)^. Lastly, Robinson *et al*.^(^^43^^)^ in an Australian cohort used a shortened EPDS and found that women in the lowest quartile of vitamin D levels (<18·8 ng/ml, similar to the deficiency level of<20 ng/ml) were at higher risk of depression at 3 d postpartum (OR 2·19 (95 % CI 1·26, 3·78); *P* = 0·006). This study ranked as having low risk of bias in five of six domains.

#### Minerals (Supplementary Appendix S2, Table S2)

Only one study assessed the relationship between Fe biomarkers and perinatal depression longitudinally. In a Spanish cohort with low risk of bias, Albacar *et al*.^(^^36^^)^ showed that Fe deficiency (ferritin <7·26 µg/l) and Fe depletion (ferritin <12 µg/l) were both associated with higher odds of developing postpartum depression at 32 weeks (Fe deficiency: OR 3·73 (95 % CI 1·84, 7·56), *P* < 0·001; Fe depletion: OR 2·30 (95 % CI 1·29, 4·10), *P* = 0·005).

An American study and a Korean study examined Fe biomarkers and perinatal depression using cross-sectional designs and both generally had a high risk of bias. Neither study found protective associations of Fe (the first did not report deficiencies and the latter found no clinical anaemia) or presented an adjusted model^(^^28^^,^^31^^)^. Two further cross-sectional studies examined Fe levels and depression in the first trimester (and also included B vitamin biomarkers). Watanabe *et al*.^(^^32^^)^ in Japan found no associations, and Lukose *et al*.^(^^29^^)^ in India found that anaemia was protective against depression in the first trimester (adjusted prevalence ratio 0·67 (95 % CI 0·47, 0·96); *P* = 0·03). The study by Lukose *et al*.^(^^29^^)^ was considered as having moderate risk of bias in three domains and low risk of bias in three domains, and Watanabe *et al*.^(^^32^^)^ only achieved a low risk of bias score in two of six domains.

A research team from Iran conducted a randomised trial supplementing women daily with Se for 6 months of pregnancy. They showed significantly higher Se blood levels and on average two points lower EPDS scores in the supplementation group (supplementation mean EPDS score: 8·8 (sd 5·1) *v.* placebo EPDS score: 10·7 (sd 4·4); *P* < 0·05)^(^^37^^)^. This study, however, had a high risk of bias due to incomplete outcome data (51 % completion) and unclear risk of bias in three other domains. Lastly, a team in Poland enrolled a small cohort of pregnant women (*n* 66), supplemented them with Zn and Mg and found a weak linear correlation between EPDS scores and Zn blood levels (*r* –0·2968; *P* = 0·01), but not Mg blood levels^(^^47^^)^. No adjusted model or effect size was reported and the study had high risk of bias in four out of six domains.

#### PUFA and fat (Supplementary Appendix S2, Table S3)

Three studies examined relationships between PUFA biomarkers and perinatal depression. A Brazilian cohort study showed that high *v.* low *n*-3 PUFA markers and low *v.* high *n*-6:*n*-3 ratios were protective against depression (EPA: OR 0·92 (95 % CI 0·86, 0·99); DHA: OR 0·96 (95 % CI 0·93, 0·99); DPA: OR 0·87 (95 % CI 0·77, 0·99); total *n*-3: OR 0·98 (95 % CI 0·96, 0·99); total *n*-6:*n*-3: OR 1·40 (95 % CI 1·09, 1·79)^(^^51^^)^. This study received a high risk of bias score in the study attrition domain due to high loss to follow-up with differences shown on baseline characteristics. They had low risk of bias in all other domains. Rees *et al*.^(^^39^^)^ in their case–control study with moderate to low risk of bias found that among many PUFA biomarkers, high DHA, high total *n*-3 PUFA and a low *n*-6:*n*-3 PUFA ratio were protective against depression (total *n*-3: OR 0·21 (95 % CI 0·05, 0·99, *P* = 0·05); DHA: OR 0·18 (95 % CI 0·04, 0·88, *P* = 0·03); *n*-6:*n*-3 ratio: OR 4·69 (95 % CI 1·00, 22·0, *P* = 0·05). Markhus *et al*.^(^^35^^)^ in Norway examined a range of PUFA markers in a small cohort study (*n* 42) that had high risk of bias in three domains and low risk of bias in the other three. Using linear regression, they found an association between a low omega-3 index in pregnancy and EPDS score postpartum (*β* = 0·39; *P* < 0·01). In their Brazilian cohort, Teofilo *et al*.^(^^52^^)^ measured both mean TAG and cholesterol levels (total cholesterol, LDL and HDL) and EPDS scores each trimester of pregnancy and found that higher HDL concentration was significantly associated with lower risk of antenatal depression in a linear mixed-effects model (*β* = −0·08 (95 % CI −0·157, −0·002); *P* = 0·04).

#### Multiple nutrients (Supplementary Appendix S2, Table S4)

The study by Bodnar *et al*.^(^^40^^)^ was the only one to examine a number of major nutrients simultaneously, and used principal components analysis to create nutrient groups, or factors, in an American cohort. They identified three factors in women less than 20 weeks pregnant: essential fatty acids, micronutrients and carotenoids, and used a robust clinical diagnosis of depression in the second and third trimesters of pregnancy. They did not find statistically significant results after adjustment for covariates, but did find a crude association between carotenoids and depression (unadjusted: OR 0·40 (95 % CI 0·20, 0·90), *P* = 0·02; adjusted: OR 0·80 (95 % CI 0·30, 2·10), *P* = 0·67). Other micronutrients and essential fatty acids were not protective. This study had a low risk of bias in all domains except for study attrition and confounding, due to only 73 % of participants having full assessment data and not fully accounting for history of depression. Two other studies examining multiple micronutrients included Fe and B vitamins, and their results are thus discussed in their respective nutrient categories.

## Discussion

Overall, there is inconsistent evidence for an influence of nutritional biomarker levels on perinatal depressive symptoms, with stronger evidence for certain nutrients. There is also not enough evidence to conclude that there is no link between nutrients and perinatal depression. Of twenty-four studies included, fourteen found protective effects of higher nutrient levels on perinatal depression, two studies reported higher nutrient levels were associated with a higher risk of perinatal depression, and the remaining eight studies found no association.

For vitamin D, there was inconsistent but stronger evidence for a protective effect, coming from more and higher-quality studies. Of ten studies on vitamin D, eight were prospective cohort studies, six showed significant protective effects, six had sample sizes over 200, and only two were of overall high risk of bias, one of which did not show any association. (Risk of bias scores are not meant to be collapsed, as heavy bias in even one domain can change study results. However, in the interest of interpretability, the risk of bias scores were combined into an overall bias assessment for the discussion. Any studies with high risk of bias in at least one domain were considered generally high risk of bias, if studies had low risk of bias in at least three domains with no high risk of bias scores, they were considered to have medium risk of bias, and studies with low risk of bias in four to six domains with no high risk of bias scores were considered to have overall low risk of bias.) For fats and PUFA there was some evidence for a protective effect, coming from one low risk of bias, medium-sized cohort study (*n* 238) examining HDL concentrations, and three high risk of bias studies addressing PUFA levels. Although only one of five studies examining Fe and perinatal depression reported a protective association, it was the only low risk of bias study in the group, a prospective cohort with sufficient power to detect an association (*n* 729). The remaining four studies on Fe were cross-sectional or case–control studies; one with medium sample size (*n* 365) and medium overall risk of bias and three with low sample sizes (<200) and high overall risk of bias.

Evidence on other minerals is weak due to single studies examining each distinct exposure. Three studies examined Se, Zn and Mg and found protective associations from higher nutrient levels and perinatal depression despite small sample sizes, but were of overall high risk of bias. One study with low risk of bias grouped many nutrients into factors and did not find any effect in adjusted analysis.

The evidence for a link between B vitamins and perinatal depression is also weak. However, the population source and/or methodology of studies may not have been suitable for detecting associations. Three of the four studies on B vitamins did not report the prevalence of nutrient deficiency and the fourth reported a low prevalence (<5 %). Two were cohort studies with overall low risk of bias, and two were cross-sectional and had medium or high risk of bias, one of which surprisingly found that anaemia protected from depression.

The weakness of the evidence base on this topic may thus be attributable to a paucity of studies and to methodological limitations of existing studies rather than an absence of true associations. We outline several methodological issues below which may offer some guidance for undertaking higher-quality studies in the future. Some practical methodological recommendations for future studies are presented in Table 2.

**Table 2.** Methodological recommendations for future studies

Recommendations on design and methods for future studies
1. Choose study populations with substantial nutrient deficiencies
2. Power studies sufficiently, considering prevalences of nutrient deficiencies and perinatal depression in the study area
3. Measure nutrient concentrations in blood or urine, and assess dietary intake to have both precise biochemical measures and to capture a range of nutrients and complex nutrient synergies
4. Measure both depression and nutrients at several time points to be able to assess magnitude of change and determine the direction of a possible causal relationship
5. Measure depression with a sufficient time gap after the nutritional assessment in line with the expected onset of depression after a nutritional deficiency
6. Standardise timing of measurements as nutrient concentrations are likely to fluctuate and generally diminish throughout pregnancy
7. Establish a personal and family history of depression and adjust for this variable as a potential confounder

### Exposure measurement

All studies measured nutrient biomarkers in the blood. While this can only capture a few biomarkers, it is generally a precise indicator of recent bioavailability. FFQ, on the other hand, measure whole diets and can thus capture complex synergies of ingested foods, but are imprecise and suffer from reporting bias^(^^53^^)^. Studies using FFQ were reviewed separately^(^^22^^)^. Four of the studies included in this review used both biomarkers and FFQ^(^^29^^,^^31^^,^^32^^,^^41^^)^, which can potentially provide a more complete picture of nutritional status. However, only one of these four studies was of high quality and linked diets to blood levels. The population of this study showed no nutrient deficiency and the authors found no association with perinatal depression^(^^41^^)^. More high-quality longitudinal studies on perinatal depression that measure both specific nutritional biomarkers and overall diets are needed.

The timing of exposure measurement is important for several reasons. Pregnancy and breastfeeding will deplete nutrient levels throughout the perinatal period, and levels measured at the beginning of pregnancy may thus not be comparable with nutrient levels around birth, even in the same woman^(^^54^^)^. Measuring all study participants at a similar time during pregnancy will remove this problem. Alternatively, one could control for gestational age at nutrient measurement. Time of measurement in pregnancy may be particularly important for water-soluble nutrients as their levels fluctuate more than fat-soluble ones^(^^55^^,^^56^^)^. At a population level, however, fluctuations should not be problematic as long as the timing of measurement is more or less the same in the comparison groups. Currently, critical times of nutrient depletion have not been defined and the possible relationship of nutrient losses with depression is unclear, and therefore it is not possible to determine whether across studies, different measurement times in the course of pregnancy and lactation would produce different results. More fundamentally, given the potential for reverse causality (depressive symptoms leading to poor self-care and poor diet, or less time outdoors for vitamin D), it is important to measure nutritional biomarkers with sufficient lead-time until depression outcome measurement.

### Outcome measurement

Clinical diagnosis of depression is the ‘gold standard’, but it is time-consuming and requires a trained clinician and is thus usually not feasible in large population studies^(^^10^^)^. Various screening tools have been developed that show good reliability, sensitivity and specificity depending on the cut-off point, and have been adapted to many different languages and cultural contexts^(^^57^^,^^58^^)^. For example, a cut-off point of ≥13 on the EPDS has been shown to have a sensitivity of 0·91 (95 % CI 0·84, 0·99) and a specificity of 0·91 (95 % CI 0·88, 0·94) for postpartum depression^(^^58^^)^. Only two tools exclude all somatic symptoms of pregnancy and have been validated specifically for the perinatal period: the EPDS and the Postpartum Depression Screening Scale^(^^59^^)^. Three (15 %) of the twenty-four included studies used a clinical interview (sometimes in combination with a screening tool), and nine (45 %) used the EPDS (one a shortened version), while the remaining eight (40 %) used tools that may not produce valid results in the perinatal period.

Having a history of mental disorders (including depression) is a known risk factor for perinatal depression, and antenatal depression is a risk factor for postpartum depression^(^^60^^)^. Eleven (55 %) of the twenty-four reviewed studies failed to take baseline depression or history of depression into account in their analysis. This can potentially confound the relationship of interest since previous depressive symptoms are likely to have a negative influence on self-care and diets and thus on nutritional status^(^^61^^)^. Failing to account for (recent) depressive history could thus lead to spurious associations.

### Other sources of bias

Many studies included in the present review suffered from weaknesses in design and reporting, often in more than one domain. Eleven (55 %) of the studies had limited power to detect an effect due to small sample size (<200), which makes null findings difficult to interpret. In the two case–control studies and one cross-sectional study that found an association (out of seven with these designs), reverse causality cannot be ruled out as a possible explanation. The same is true for some cohort studies where lag time between exposure and outcome measurement was very short, the lowest being 3 weeks^(^^46^^)^.

The large cohorts generally described enrolment and follow-up protocols in detail (such as Blunden *et al*.^(^^41^^)^ and Cassidy-Bushrow *et al*.^(^^33^^)^), but several of the other studies did not (such as Pinto *et al*.^(^^51^^)^), making it difficult to judge risk of bias. Thirteen studies (54 %) did not include important potential confounders, such as basic sociodemographic variables, and thus may have overestimated the strength of the association. In the special case of vitamin D, the compound can be ingested through foods or synthesised by the human body if skin is exposed to sunlight. Sunlight exposure also has a proposed direct link to depression outside the dietary intake pathway^(^^62^^)^. Hence, any nutritional analysis of vitamin D should adjust for sunlight exposure or a proxy thereof. Lastly, statistical model building and reporting was unclear in some studies, which made it difficult to interpret the results and assess the evidence.

Most of these studies were observational (since blood nutrient concentrations cannot be directly increased) and suffered from several limitations, which can explain some inconsistencies in the results. A recent review on the relationship between dietary intake and supplementation with perinatal depression similarly found that there was inconclusive evidence of an association between certain diets and foods with perinatal depression. The strongest PUFA experimental study in the previous review found no effect on depression, but other trials also on PUFA did find an association. Taken together, the evidence from both reviews suggests that, although not definitive, higher *v.* lower concentrations of PUFA may be protective against depression^(^^22^^)^, but would require further investigation. Most trials focus on one nutrient or supplement combination, rather than diets as a whole. In the previous review on diets and supplementation, observational studies also generally found a protective effect from healthier or Mediterranean diets as a whole^(^^22^^)^.

### Prevalence of nutritional deficiency

Sixteen of twenty-four studies included in the present review are from high-income countries where nutrient deficiencies are relatively rare^(^^56^^)^. Three others were undertaken in Turkey, Brazil and Iran, which are upper-middle-income countries^(^^37^^,^^48^^,^^52^^)^, and one study came from India, a lower-middle-income country^(^^29^^)^. In higher-income settings, there may be insufficient variation in nutrient levels and too few participants in the clinically deficient range to detect an association. For example, Blunden *et al*.^(^^41^^)^ reported no associations with perinatal depression when examining folate in a setting where 96 % of the women reported taking folic acid supplements during pregnancy, and the prevalence of folate deficiency was below 5 %. Studies from low-income countries, where nutritional deficiencies are still widespread and more severe, would therefore have a much better chance to establish this relationship^(^^2^^,^^5^^)^.

Vitamin D deficiency is common in many populations around the world, including in high-income countries^(^^63^^)^. Insufficiency in the study populations of the reviewed articles ranged from 24 %^(^^43^^)^ to 85 %^(^^44^^)^. Higher prevalence of deficiency provided more power to detect an association with perinatal depression, and indeed, for vitamin D, the evidence of an association with perinatal depression was most convincing. There is also evidence in the literature of an association between vitamin D and depression in general, i.e. not just in the perinatal period^(^^64^^)^.

### Practical implications

While there is an emerging body of evidence on the treatment of perinatal depression with PUFA^(^^65^^)^, there has been little research so far on prevention. Prevention strategies can be broader than treatment strategies, as they work ‘upstream’ of the problem and can address several risk factors and outcomes concomitantly. The *Lancet* series on perinatal mental health in 2014 highlighted risk factors for perinatal depression including low socio-economic status, trauma, domestic violence, lack of support, migration status, history of psychopathology, and chronic illness and medical problems^(^^66^^)^. Some treatment options (and to a lesser extent prevention strategies) proposed in LMIC are embedded within complex interventions that have health and economic components related to nutrition^(^^13^^,^^66^^)^. Understanding the root causes of perinatal depression, including the role of nutrition, may encourage a shift from treatment to prevention, and targeting these root causes might simultaneously alleviate other adverse health outcomes. Developing prevention strategies could also reduce the need for treatment options, which are often inaccessible to women in LMIC^(^^67^^)^.

### Strengths and limitations

We gathered all available evidence on whether blood levels of different nutrients are associated with perinatal depression. We considered both antenatal and postpartum depression, which traditionally have been separated^(^^8^^,^^30^^)^. Previously, the focus has been on postpartum depression, but evidence is emerging that antenatal depression is both common unto itself and a risk factor for postpartum depression^(^^68^^–^^70^^)^ and should thus no longer be ignored. Because of the heterogeneity of both the exposure and the outcome classification, a meta-analysis was not possible, but we provide a narrative synthesis of evidence on the topic.

A limitation of the present review is that it may suffer from publication bias. Studies finding null results are less likely to be published in English-language scientific journals and we did not include other languages or grey literature. However, we did identify six studies that reported no significant associations (B vitamins, vitamin D, Fe, and all nutrients grouped in principal components analysis) and two studies that reported high nutrient levels (B vitamins) or anaemia as risk factors for perinatal depression, contrary to expectation. Furthermore, there may be many more unknown relationships between nutrients other than those in the present review and perinatal depression, which have yet to be examined and thus are not part of this synthesis.

## Conclusions

This synthesis of available evidence suggests that blood levels of certain nutrients potentially play a role in the development of perinatal depression, but results are inconsistent and we lack confidence in effect estimates. The evidence appears to be stronger for vitamin D compared with B vitamins or minerals. However, many studies exhibited serious methodological limitations, and in several nutrient categories only one study of poor quality was available, reducing the ability to draw robust conclusions. These findings are similar to what we found when reviewing studies on the influence of dietary intake and supplementation on perinatal depression. While overall inconclusive, there was some evidence that perinatal depression is linked to certain diets and foods^(^^22^^)^. To strengthen the evidence base on this topic, robust longitudinal studies are needed from settings with higher prevalence of nutritional deficiencies that ideally measure both specific nutritional biomarkers and overall diets.

## Supplementary material

For supplementary material accompanying this paper visit http://dx.doi.org/10.1017/jns.2017.58. click here to view supplementary material
