# Supplementary material for: Nutrients and perinatal depression: a systematic review
Source: J Nutr Sci. 2017 Dec 20;6:e61. doi: 10.1017/jns.2017.58 (PMC5738654; doi:10.1017/jns.2017.58)
Supplement: Supplementary file 1 [file S2048679017000581sup001.docx]

**Supplementary Appendix S1. Risk of bias assessment**

We assessed the confounding domain based on the potential confounders controlled for and the method by which this was done. Several socioeconomic and psychosocial variables are related to both depression and self-care or eating habits (and thus nutritional status). In particular, accounting for a history of depression, marital status or social support, general health status, income and employment ^(1)^ is likely to reduce confounding. Only studies that accounted for several of these variables, including a baseline measure or history of depression, were considered as having a low risk of bias, studies that did not control for any of these variables were considered at high risk of bias. Accounting for seasonality or sunlight exposure, a main source of Vitamin D, was required to be ranked as having low risk of bias in studies on Vitamin D. Most studies dealt with confounding in stepwise models, conducting a bivariable analysis as a first step to identify potential confounders. If studies included important potential confounders in a crude model and found that these were not associated with the outcome, and therefore did not include them in their final adjusted model, we considered this sufficient control for confounding in our risk of bias assessment.

For the domain on statistical analysis and reporting, we assigned low risk of bias if studies included important potential confounders using a step-by-step approach, but did not proceed to a fully adjusted multivariable model because they found no association between the exposure(s) of interest and the outcome. However, if authors did not attempt an adjusted model despite significant associations in a crude model or their analysis strategy was unclear, we ranked studies as having moderate or high risk of bias in the domain on statistical analysis and reporting.

**Supplementary Appendix S2. Supplementary Results tables**

| **Supplementary Appendix S2, Table S1: Results of studies evaluating associations between vitamins and perinatal depression** | | | | | | | | | |
| --- | --- | --- | --- | --- | --- | --- | --- | --- | --- |
| **First author (Year)** | **n** | **Nutrients analyzed** | **Main Analysis** | **Unit of Exposure** | **Unit of outcome** | **Unadjusted Associations significant at p<0.10 (effect size, 95% CI, p value)** | **Adjusted Associations significant at p<0.10  (effect size, 95% CI, p value)** | **Potential confounders considered** | **Limitations** |
| **B VITAMINS** | | |  |  |  |  |  |  |  |
| Blunden (2012) | 2,856 | Plasma folate concentrations | Multivariable Poisson regression (nutrients only univariable) | Geometric means compared between depressed and non-depressed group | EPDS score ≥13 vs. <13 | Red-cell folate not significant | Not reported | History of mental illness*; education; scoring positive for depression on the GHQ*; smoking; alcohol; social benefits; financial strain; breastfeeding*; folic acid supplements; Folate intake, Vitamin B6 intake, Vit B12 intake | • Less than 5% of study population with low/marginal folate • PPD screening done on average at 1 y PP |
| Chong (2014) | 709 | Plasma concentrations (ng/mL): folate, vitamin B12 | Multivariable logistic regression | Means, quartiles (ng/mL): Q1: 1.5—9.7  Q2: 9.7—13.7 Q3: 13.7—17.8 Q4: 17.8—277 | Mean differences in Vit D concentrations between EPDS score ≥12 vs.<12: • 1 w PP: means NR, p=0.003 • 6 w PP: means NR, p=0.004 • 6 m PP: means NR, p<0.001 Correlation between Vit D level and EPDS points: • 1 w PP: r = -0.2, p=0.02 • 6 w PP: r = -0.2, p=0.01 • 6 m PP: r = -0.3, p<0.01 | Antenatal EPDS score ≥15 vs.<15:  • Folate: 27.3 (SD 13.8) vs. 40.4 (SD 36.5), p=0.011 • Vitamin B12: NS Postpartum EPDS score ≥13 vs.<13: Not shown | Antenatal EPDS score ≥15 vs.<15: • Folate: OR 0.69 per SD increase (CI 0.52--0.94), p=0.016 Postnatal EPDS score ≥13 vs.<13:  • Folate: OR 0.75 per SD increase (CI 0.58--0.99), p=0.04; adjusted for antenatal depression OR 0.84 (CI 0.62--1.12, p=0.25) • Vitamin B12: NS at either time point | Age, education, ethnicity, gravidity, obstetric and neonatal complications, smoking and smoke exposure, alcohol consumption, marital status, pre-pregnancy BMI, folic acid and Vit B12 supplementation, history of depression | • High loss to follow-up (74% complete at 3 m) • Prevalence of nutrient deficiencies not reported |
| **VITAMIN D** | | | |  |  |  |  |  |  |
| Accortt (2015) | 91 | Serum 25-hydroxyvitamin D3 concentrations | Multivariable linear regression | Continuous, transformed log 25-OHD concentrations | EPDS score; linear | Linear EPDS score: β --0.145 (CI NR), p=0.17 | Linear EPDS score: β --0.209 (CI NR), p=0.058 | Age, marital status, education, employment status, income, smoking, pre-pregnancy BMI, parity, history of hypertension, history of preterm birth, preterm birth, LBW, prenatal CES-D score, pre-pregnancy history of depression, season, inflammatory markers | • High loss to follow-up (48% completion) |
| Brandenbarg (2012) | 4,101 | Serum 25-hydroxyvitamin D3 concentrations | Multivariable logistic regression | Clinical strata (ng/mL): Normal (≥32) (Reference) Sufficient (20-32) Insufficient (12-<20) Deficient (≤ 12); Log odds per 4 ng/mL decrease in Vit D | CES-D ≥ 16 vs. <16 | CES-D score ≥16 vs.<16:  Normal: Reference Sufficient: OR 1.37 (CI 1.11--1.67), p=0.002 Insufficient: OR 2.13 (CI 1.69--2.68), p<0.001 Deficient: OR 3.36 (CI 2.68--4.21), p<0.001 | CES-D score ≥16 vs.<16:  Normal: Reference Sufficient: OR 1.21 (CI 0.97--1.51), p=0.09 Insufficient: OR 1.44 (CI 1.12--1.85), p=0.004 Deficient: OR 1.48 (CI 1.13--1.95), p=0.005 Linear trend: OR 1.05 (CI 1.02--1.08), p<0.001 Interaction between season of blood sample and Vit D: p=0.39 | Age*, parity*, ethnicity*, pre-pregnancy BMI*, smoking*, alcohol consumption*, desirability of the pregnancy*, education*, cohabitation*, employment*, seasonal fluctuation* | • Short lag time between exposure and outcome (13 w to 16 w) • History of depression not included |
| Cassidy-Bushrow (2012) | 178 | Serum 25-hydroxyvitamin D3 concentrations | Multivariable logistic regression | Continuous, transformed log 25-OHD concentrations | CES-D ≥16 vs. <16 | Log odds per 1 ng/mL increase: 0.49 (CI 0.28--0.87), p=0.014 | Log odds per 1 ng/mL increase: 0.54 (CI 0.29--0.99), p=0.046 | Age*, marital status*, education*, employment, income, smoking, alcohol use, pre-pregnancy BMI, history of hypertension, type II diabetes, gestational age, parity, history of preterm birth, history of depressive illness, prenatal vitamin and Vit D supplementation, seasonal fluctuation*, lag time between exposure and outcome* | • Short lag time between exposure and outcome (avg. 9 w to avg. 20 w) • High non-participation (203 out of 652 enrolled) • Study population only African American women |
| Fu (2014) | 213 | Serum 25-hydroxyvitamin D3 concentrations | Multivariable logistic regression | Clinical strata (ng/mL): Deficient: <20  Insufficient: 20–30  Normal: >30 | EPDS score ≥12 vs. <12 | Correlation of Vit D concentration and EPDS score:  • r = -0.293, P < 0.001 EPDS score ≥12 vs. <12: • OR 0.74 (CI 0.64–0.85), p < 0.001 | EPDS score ≥12 vs. <12: • OR 0.81 (CI 0.70–0.92), p < 0.001 per Vit D strata | Age, marital status, employment, ethnicity, parity, gravidity, alcohol use, smoking, birth weight, BMI, breastfeeding, stressful life events*, maternal education, family income, partner support*, planned vs. unplanned pregnancy, mode of delivery and previous psychiatric contact* | • Medium loss to follow-up (86% completion) |
| Gould (2015) | 1040 | Serum 25-hydroxyvitamin D3 concentrations (within PUFA trial) | Multivariable logistic regression | Three concentration levels in cord blood (ng/mL):  L1: <10  L2: 10-20  L3: >20 | EPDS score ≥12 vs. <12 | EPDS score ≥12 vs.<12: Linear trend (per nmol/L): • Overall, 6 w: RR 0.91 (CI 0.84--0.99), p=0.05 • Overall, 6 m: RR 0.94 (CI 0.86--1.01), p=0.21 Categorical (L1: Reference): • Overall, 6 m:  L2: RR 0.71 (CI 0.38--1.30) L3: RR 0.61 (CI 0.34--1.10) • Control group, 6 w:  L2: RR 0.40 (CI 0.22--0.74) L3: RR 0.24 (CI 0.13--0.45) • Intervention group, 6 w:  L2: RR 0.93 (CI 0.28--3.10) L3: RR 1.19 (CI 0.38--3.73) | EPDS score ≥12 vs.<12: Linear trend (per nmol/L): • Overall, 6 w: RR 0.92 (CI 0.84–1.02), p=0.11 • Overall, 6 m: RR 0.96 (CI 0.88–1.05), p=0.41 Categorical (L1: Reference): • Overall, 6 m:  L2: RR 0.84 (CI 0.46--1.56) L3: RR 0.84 (CI 0.45--1.58) • Control group, 6 w:  L2: RR 0.35 (CI 0.17--0.69) L3: RR 0.24 (CI 0.12--0.51)  • Intervention group, 6 w:  L2: RR 2.01 (CI 0.55--7.30) L3 RR 2.19 (CI 0.65--7.40) | Treatment group (of PUFA trial), race, age, parity, BMI, supplements, smoking, center enrolled, MSSI score | • Cord blood measurement of Vit D not standard, only proxy |
| Gur (2014) | 189 | Serum 25-hydroxyvitamin D3 concentrations | Mean differences, Pearson correlations | Continuous, comparison of means, correlations | EPDS score ≥12 vs. <12 | Mean difference in Vit D concentration between EPDS score ≥12 vs.<12: • 1 w PP: means NR, p=0.003 • 6 w PP: means NR, p=0.004 • 6 m PP: means NR, p<0.001 Correlation between Vit D concentration and EPDS score: • 1 w PP: r = -0.2, p=0.02 • 6 w PP: r = -0.2, p=0.01 • 6 m PP: r = -0.3, p<0.01 | Not reported | Potential confounders not measured, but sample restricted to women with following characteristics: married, desired pregnancy, BMI 20–30, parity <3, education >8 years, income >US $4500, Caucasian, age 18–40, non-employed, non-smoker, non-alcohol drinker, no known medical or psychiatric disease, single pregnancy, and native Turkish speaker. | • No adjusted model of the association of interest • Seasonality or sun exposure not included • Unclear statistical reporting • Restriction led to small sample size |
| Murphy (2010) | 97 | Serum 25-hydroxyvitamin D3 concentrations | Multivariable linear mixed model | Clinical strata (ng/mL): Insufficient (≤32) vs. Sufficient (>32) | Mean score of 7 measurements of EPDS score ≥9 vs. <9 | Mean EPDS sum scores between strata:  3.71 (SE 0.24) vs. 3.38 (SE 0.24), p=0.27 | Mean EPDS sum scores between strata: 3.68 (SE 0.30) vs. 2.92 (SE 0.32), p=0.021 | Age, race/ethnicity, gender, marital status, insurance status, season, feeding method, Vit D dose (from trial), planned pregnancy | • Convenience sample  • Sum score calculation not explained • History of depression not included |
| Robinson (2014) | 796 | Serum 25-hydroxyvitamin D3 concentrations | Multivariable linear and logistic regression | Quartiles (ng/mL): Q1: <20 Q2: 20–23 Q3: 24–28 Q4: >28 (Ref) Clinical cut-point for deficiency: <20 ng/mL | Shortened EPDS; 6 | % of women with 6+ symptoms in  Q1: 27% Q2: 20% Q3: 23% Q4: 17% p for trend: 0.017 | Quartiles of Vit D concentrations and linear EPDS score: Q1: β 0.93 (CI 0.27--1.58), p=0.005 Q2: β 0.12 (CI −0.53--0.77), p=0.721  Q3: β 0.38 (CI −0.26--1.03), p=0.245  Q4: Reference Logistic regression of short EPDS ≥6 vs. <6: Q1: OR 2.19 (CI 1.26--3.78), p=0.006 Q2: OR 1.42 (CI 0.80--2.54), p=0.236  Q3: OR 1.52 (CI 0.85--2.72), p=0.158  Q4: Reference | Age, education, total family income, hypertensive diseases of pregnancy, gender of child, admission to the Special Care Nursery, proportion of birth weight, pre-pregnancy BMI, smoking, alcohol use, season of birth | • PPD screening tool not standard or validated  • History of depression not included |
| Huang (2014) | 498 | Serum 25-hydroxyvitamin D3 concentrations | Multivariable linear and logistic regression | Continuous, quartiles (ng/mL): Q1: ≥39.5 (Ref) Q2: 34--39.4 Q3: 28.9--34 Q4: <28.9 | DASS-21, linear PHQ-9, linear | DASS 21 Depression score: NS PHQ-9: Continuous Vit D level: β 0.040 (CI 0.004--0.077), p=0.029 Q1: Reference Q2: β 0.32 (CI -0.52--1.16) Q3: β -0.51 (CI -1.34--0.33) Q4: β 1.11 (CI 0.20--2.02), p<0.05 P for trend: 0.083 | DASS 21 Depression score, NS PHQ-9: Continuous Vit D level: β 0.019 (CI -0.020--0.058), p=0.34 Q1: Reference Q2: β 0.09 (CI -0.79--0.98) Q3: β -0.74 (CI -1.64--0.15) Q4: β 0.55 (CI -0.42--1.52) P for trend: 0.656 | Season, gestational age, age at enrollment, pre-pregnancy BMI, smoking, race, education, and marital status | • PPD screening tool not standard • History of depression not included |
| Nielsen (2013) | 1,480 | Serum 25-hydroxyvitamin D3 concentrations | Multivariable logistic regression | Six concentration levels (ng/mL): Level 1: <6  Level 2: 6–9.9 Level 3: 10–19.9 Level 4: 20-31.9 (Ref) Level 5: 32–39.9 Level 6: ≥40 | Rx for antidepressants w/o admission to hospital within 1 y PP vs. no Rx within 1 y PP | Rx vs. no Rx: L1: OR 1.70 (CI 0.91--3.16) L2: OR 1.05 (CI 0.70--1.58) L3: OR 1.26 (CI 0.98--1.61) L4: Reference L5: OR 1.30 (CI 0.93--1.82) L6: OR 1.77 (CI 1.07--2.93) | Rx vs. no Rx: L1: OR 1.35 (CI 0.64--2.85), p=0.43 L2: OR 0.83 (CI 0.50--1.39), p=0.48 L3: OR 1.13 (CI 0.84--1.51), p=0.82 L4: Reference L5: OR 1.53 (CI 1.04--2.26), p=0.03 L6: OR 1.89 (CI 1.06--3.37), p=0.03 p=0.08 (test for homogeneity) | Season, gestational week of sampling, parity, smoking, socioeconomic status, pre-pregnancy BMI, physical activity, social support, multivitamin supplement | • Rx for depression used as proxy for diagnosis, which excludes undiagnosed and subclinical cases • Prevalence of nutrient deficiencies not reported |
| • Depression terms: Admit: admission to hospital; CES-D = Center for Epidemiological Studies Depression Scale; DASS 21: Depression, Anxiety, and Stress Scales; EPDS = Edinburgh Postpartum Depression Scale; EPDS-V: Edinburgh Postpartum Depression Scale for Vietnam; GHQ: General Health Questionnaire; MSSI: Mainz Severity Scoring Index; PD: perinatal depression; PP: Postpartum; PPD: Postpartum depression; PHQ-9: Patient Health Questionnaire Depression Module; Rx: prescription; SCL-90-R = Symptom Checklist-90-Revised  • Measurement terms: avg: Average; d: day(s); m: month(s); tri: trimester(s); w: week(s); y: year(s); g/dl: grams per deciliter; L: level; mg/l: milligrams per liter; μg/l: micrograms per liter; μmol: millimole • Statistical terms: *Significant (p < 0.05) in final model; CI: confidence interval; NR: Not reported; NS: not significant; OR: odds ratio; Q: quartile; Ref: Reference; SD: Standard deviation; SE: standard error  • Nutrient terms: 25-OHD: Serum 25-hydroxyvitamin D3 concentrations; BMI: Body Mass Index; LBW: low birth weight; PUFA: Polyunsaturated Fatty Acid; Vit: Vitamin(s) | | | | | | | | | |

| **Supplementary Appendix S2, Table S2: Results of studies evaluating associations between minerals and perinatal depression** | | | | | | | | | |
| --- | --- | --- | --- | --- | --- | --- | --- | --- | --- |
| **First author (Year)** | **n** | **Nutrients analyzed** | **Main Analysis** | **Unit of Exposure** | **Unit of outcome** | **Unadjusted Associations significant at p<0.10 (effect size, 95% CI, p value)** | **Adjusted Associations significant at p<0.10 (effect size, 95% CI, p value)** | **Potential confounders considered** | **Limitations** |
| **IRON** |  |  |  |  |  |  |  |  |  |
| Albacar (2011) | 729 | Serum concentrations:  Fer (μg/L), Tf (g/L), Iron (μmol/L), CRP (mg/L), TfS (%) | Multivariable logistic regression | Iron depletion: Fer <12 Marginal iron deficiency: Fer <12 and TfS <16%  Iron deficiency: Fer <7.26 | Screened with EPDS, >9 diagnosed with SCID-CV | Clinically diagnosed depressed vs. non-depressed: • Ferritin: 15.4 vs. 21.6, p<0.01 • Tf: 2.9 vs. 2.8, p=0.51 • Iron: 9.3 vs. 8.8, p=0.39 • TfS: 16.1 vs. 14.9, p=0.32 • Depletion of iron stores: 38.5% vs. 23.3%, p=0.01 • Marginal iron deficiency: 15.6% vs. 13.7%, p=0.68 | Clinically diagnosed depressed vs. non-depressed: Iron depletion:  • Ferritin: OR 2.30, (CI 1.29–4.10), p<0.01 • TfS: OR 0.77 (CI 0.45–1.31), p=0.34 Iron deficiency:  • Ferritin: OR 3.73 (CI 1.84–7.56), p=<0.01 • TfS: OR 0.72 (0.42–1.23), p=0.23 | Age, marital status, employment*, education, breastfeeding, caesarean delivery, parity, CRP | • History of depression not included |
| Aubuchon-Endsley (2012) | 82 | Serum concentrations: Hb (g/dL), sTfR (mg/L), Fer (μg/L), AGP | Spearman's rank correlations | Linear correlations | Continuous SCL-90-R score | Correlation of nutrient and SCL score: • Iron intake: r= 0.12, p=0.24 • Hb: r= -0.01, p=0.92 • sTfR: r= 0.11, p=0.27 • Fer: r= -0.11, p=0.32 | Not reported | Reported no associations between outcome and age, race/ethnicity, education, employment or income; data not shown | • PPD screening tool not standard • Low prevalence and severity of depressive symptoms • No iron deficiency in study population • No adjusted model of the association of interest • Primary study outcome not depression • History of depression not included |
| Bae (2010) | 114 | Plasma concentrations: WBC (10³/μL) RBC (106/μL), HbG (g/dL), Hct (%), MCV (fl), MCH (pg), MCHC (g/dl), RCDW (%), PLT (10³/μL), PDW, MPV (fl) | T-test, chi-squared test | Mean nutrient concentrations | BDI score ≥10 vs. <10 | BDI score ≥10 vs. <10: • All measures not significant | Not reported | Tested for association with the outcome, but not included in final model: age, pre-pregnancy BMI, pregnancy BMI, delivery birth BMI, parity*, education, income, occupation, morning sickness, nutritional supplements | • Cut-point for depression based on mean, not validated cut-point for screening depressed vs. non-depressed • Assessment timing not standard for all participants • History of depression not included • No adjusted model of the association of interest |
| **SELENIUM** |  |  |  |  |  |  |  |  |  |
| Mokhber (2011) | 166 | Group A: 100 mg selenium per day Group B: Placebo | T-test, chi-squared test | Selenium vs. placebo group | EPDS score; mean difference | Supplement vs. placebo group: • 8.8 (SD 5.1) vs. 10.7 (SD 4.4), p<0.05 (Serum selenium concentrations in supplement vs. placebo group: • 168.6 (SD 36.4) vs. 119.4 (SD 33.4), p<0.001) | Not reported | No baseline differences in age, gestational age, social support score, smoking, alcohol use, education, family medical and psychological history, thyroid function | • High loss to follow-up (51% completion) • Adherence to supplementation low • Unclear statistical reporting • Prevalence of nutrient deficiencies before supplementation not reported |
| **ZINC and MAGNESIUM** | | |  |  |  |  |  |  |  |
| Wojcik (2006) | 66 | Serum concentrations of Zn and Mg in cohort of pregnant women given supplements: 25 mg Zn per d (4 m until delivery), 470 mg magnesium lactate + Vit B6 2-3 times/d (7 m until delivery). | T-test, ANOVA, Spearman's rank correlations | Linear correlations | Continuous EPDS score | Correlation of Zn and EPDS score: (including assessment at both 3 d and 30 d PP): • r = –0.2968, p = 0.014 Correlation of Mg and EPDS score: • NS at either time point | Not reported | None included, but baseline sample limited to 31 ± 1 years old, in good physical health, without history of psychotic or affective disorder | • No adjusted model of the association of interest • High loss to follow-up (41% completion) • No comparison group • Unclear statistical reporting • Sample made homogenous instead of including covariates in model • Prevalence of nutrient deficiencies not reported |
| • Depression terms: BDI = Beck's Depression Inventory; EPDS = Edinburgh Postpartum Depression Scale; PD: perinatal depression; PP: Postpartum; PPD: Postpartum depression; SCID-CV: structured clinical interview for depression; SCL-90-R = Symptom Checklist-90-Revised  • Measurement terms: avg: Average; d: day(s); m: month(s); tri: trimester(s); w: week(s); y: year(s); fl: fluid liters; g: grams; g/dl: grams per deciliter; L: level; mg/l: milligrams per liter; μl:microliters; μg/l: micrograms per liter; μmol: millimole; nmol: nanomole  • Statistical terms: *Significant (p < 0.05) in final model; ANOVA: Analysis of variance; CI: confidence interval; NR: Not reported; NS: not significant; SD: Standard deviation; SE: standard error  • Nutrient terms: AGP: inflammatory marker 1-acid glycoprotein; BMI: Body Mass Index; CRP: inflammatory marker cardiac C-Reactive Protein; Fer: ferritin; Hb: Hemoglobin; Hct: Hematocrit; IDA: Iron deficiency anemia; MCH mean corpuscular hemoglobin; MCHC: mean corpuscular hemoglobin concentration; MCV: mean corpuscular volume; Mg: Magnesium; MPV: mean platelet volume; PDW: platelet distribution width; PLT: platelet; RBC: red blood cell; RCDC: red cell distribution width; sTfR: soluble transferrin receptors; Tf: transferrin; TfS: free iron and transferrin saturation; Vit: Vitamin(s); WBC: white blood cell; Zn: Zinc | | | | | | | | | |

| **Supplementary Appendix S2, Table S3: Results of studies evaluating associations between fat and fatty acids and perinatal depression** | | | | | | | | | |
| --- | --- | --- | --- | --- | --- | --- | --- | --- | --- |
| **First author (Year)** | **n** | **Nutrients analyzed** | **Main Analysis** | **Unit of exposure** | **Unit of outcome** | **Unadjusted Associations significant at p<0.10 (effect size, 95% CI, p value)** | **Adjusted Associations significant at p<0.10  (effect size, 95% CI, p value)** | **Potential confounders considered** | **Limitations** |
| Markhus (2013) | 43 | Serum concentrations (μg/ml): DPA, DHA, n-3 index^1^, n-6:n-3 ratio, total HUFA score^2^, n-3 HUFA score (mg/l) | Univariable linear regression, Pearson correlations | Linear nutrient concentrations, ratios | EPDS score; linear | Correlation of nutrient and EPDS score: • n-3 index: r= -0.39, p=0.01 • n-3/n-6 ratio: r= -0.31, p=0.04 • Total HUFA score: r= -0.35, p=0.02 • n-3 HUFA score: r= -0.32, p=0.04 | Not reported | Correlation coefficients measured for age, education, income, emotional distress*, negative life events, partner satisfaction, and social support from friends/family, but not included in model | • High loss to follow-up (50% completion) • Selection bias: higher EPDS scores and lower n-3 status in non-completers than completers • History of depression not included • No adjusted model of the association of interest |
| Teofilo (2014) | 238 | Serum concentrations (mg/dl):  Triglycerides, total cholesterol, LDL, HDL | Multivariable linear regression | Tertiles of serum concentration and linear increase | EPDS score ≥11 vs. <11 | Linear EPDS score: • Triglycerides: NS • Total cholesterol: NS • LDL: NS • HDL: β= -0.079 (CI -0.157 – -0.002), p=0.045 | Linear EPDS score: • HDL: β= -0.08 (CI -0.157 – -0.002), p=0.043 | Age*, education*, gestational age*, parity*, marital status*, physical activity*, work outside home*, unplanned pregnancy*, pre-pregnancy BMI, anxiety disorder*, suicidal ideation* and physical violence* | • History of depression not included • Prevalence of nutrient deficiencies not reported |
| Pinto (2017) | 172 | Serum concentrations (μg/ml): ALA, EPA, DPA, DHA, LA, AA, γ LA, EDA, ETE, total n-6:n-3 ratio | Multivariable logistic regression, predictive probability curves | Linear nutrient concentrations, ratios | EPDS score ≥11 vs. <11 | EPDS score ≥11 vs. <11: • ALA: OR 0.93 (0.89–0.97) • EPA: OR 0.94 (0.88–1.01) • DHA: OR 0.95 (0.93–0.98) • DPA: OR 0.88 (0.77–0.99) • Total n-3: OR 0.97 (0.96–0.98) • Total n-6:n-3 ratio: OR 1.13 (0.91–1.42) • LA, AA, LA, EDA, ETE (all n-6 PUFA): NS | EPDS score ≥11 vs. <11: • EPA: OR 0.92 (CI 0.86–0.99), p<0.05 • DHA: OR 0.96 (CI 0.93–0.99), p<0.05 • DPA: OR 0.87 (CI 0.77–0.99), p<0.05 • Total n-3: OR 0.98 (CI 0.96–0.99), p<0.05 • Total n-6:n-3 ratio: OR 1.40 (CI 1.09–1.79), p<0.05 • ALA, LA, AA, LA, EDA, ETE: NS | Age, schooling, possible and diagnosed previous history of depression, marital status, smoking, alcohol consumption, family income, parity, planned pregnancy, BMI | • High loss to follow-up (79% completion) • Prevalence of nutrient deficiencies not reported |
| Rees (2009) | 38 | Serum concentrations (μg/ml): n-3 PUFA, DHA, EPA, ALA, n-6 PUFA, LA, AA, DPA, n-6:n-3 ratio, AA:EPA, DHA:DPA | Pearson correlations, multivariable logistic regression | High vs. low (split at median) | Screened with EPDS, >9 diagnosed with SCID-CV | Clinically diagnosed depressed vs. non-depressed: • Total n-3: OR 0.16 (CI 0.04–0.66), p=0.01 • DHA: OR 0.16 (CI 0.04–0.66), p=0.01 • n-6:n-3 ratio: OR 6.45 (CI 1.42–27.24), p=0.01 • ALA, EPA, total n-6, AA, DPA, AA:EPA ratio, DHA status (DHA:DPA ratio): NS | Clinically diagnosed depressed vs. non-depressed: • Total n-3: OR 0.21 (CI 0.05–0.99), p=0.05 • DHA: OR 0.18 (CI 0.04–0.88), p=0.03 • n-6:n-3 ratio: OR 4.69 (CI 1.00–21.99), p=0.05 • ALA, EPA, total n-6, AA, DPA, AA:EPA ratio, DHA status (DHA:DPA ratio): NS | Age*, education*, parity*, marital status, smoking, baseline EPDS score | • Unclear whether cases and controls recruited from same population • Prevalence of nutrient deficiencies not reported |
| 1: n-3 index: The content of EPA+DHA in red blood cells membranes expressed as a percent of total fatty acid 2: Total HUFA is the sum of the omega-3 and the omega-6 HUFAs, and the red blood cells omega-3 HUFA score equals 100% - omega-6 HUFA • Depression terms: EPDS = Edinburgh Postpartum Depression Scale; PPD: Postpartum depression; SCID-CV: structured clinical interview for depression • Measurement terms: avg: Average; l: liters; g: grams; g/dl: grams per deciliter; mg/l: milligrams per liter; μg/l: micrograms per liter; μmol: millimole • Statistical terms: *Significant (p < 0.05) in final model; CI: confidence interval; NR: Not reported; NS: not significant; SD: Standard deviation • Nutrient terms: AA: arachidonic acid; AdA: docosatetraenoic acid; ALA: alpha-linolenic acid; BMI: Body Mass Index; DHA: Docosahexaenoic acid; DPA: docosapentaenoic acid; EDA: eicosadienoic acid; EPA: Eicosapentaenoic acid; ETE: eicosatrienoic acid; HDL: high-density lipoproteins; HUFA: Highly unsaturated fatty acids; LA: linolenic acid; LDL: Low-density lipoproteins; n-3 PUFA: omega-3 polyunsaturated fatty acids; n-6 PUFA: omega-6 polyunsaturated fatty acids; PUFA: Polyunsaturated Fatty Acid | | | | | | | | | |

| **Supplementary Appendix S2, Table S4: Results of studies evaluating associations between multiple nutrients and perinatal depression** | | | | | | | | | |
| --- | --- | --- | --- | --- | --- | --- | --- | --- | --- |
| **First author (Year)** | **n** | **Nutrients analyzed** | **Main Analysis** | **Unit of Exposure** | **Unit of outcome** | **Unadjusted Associations significant at p<0.10 (effect size, 95% CI, p value)** | **Adjusted Associations significant at p<0.10 (effect size, 95% CI, p value)** | **Potential confounders considered** | **Limitations** |
| **ALL NUTRIENTS** | | |  |  |  |  |  |  |  |
| Bodnar (2012) | 135 | Nutrient concentrations: Red cell AA (% weight), red cell EPA (% weight), red cell DHA (% weight), plasma folate (nmol/l), Hcy (μmol/l), plasma Vit C (nmol/l), Vit D (nmol/l), Vit A (nmol/l), serum α-tocopherol (nmol/l), serum β-carotene (nmol/l), serum lutein+zeaxanthin (nmol/l), serum b-cryptoxanthin (nmol/l), serum lycopene (nmol/l), Fer, sTfR (μmol/l) | PCA and multivariable logistic regression | Tertiles of factor 1 (EFA), factor 2 (micronutrients), factor 3 (carotenoids) | Clinically diagnosed depressed vs. non-depressed | Low vs. high tertile of factor score: Factor 1 (EFA): NS Factor 2 (micronutrients): NS Factor 3 (carotenoids):  • T1 (lowest): Reference • T2: OR 0.4 (CI 0.2-0.9), p=0.017 • T3 (highest): OR 0.4 (CI 0.2-0.9), p=0.017 | Low vs. high tertile of factor score: Factor 1 (EFA): NS Factor 2 (micronutrients): NS Factor 3 (carotenoids):  • T1 (lowest): Reference • T2: OR 0.5 (CI 0.2-1.4), p=0.16 • T3 (highest): OR 0.8 (CI 0.3-2.1), p=0.67 | Race/ethnicity, pre-pregnancy BMI*, maternal age, education*, marital status, work status*, parity, anti-depressant use and season | • No nutrient deficiencies detected in study population • Included women with and without depression at baseline |
| **IRON and B VITAMINS** | | |  |  |  |  |  |  |  |
| Lukose (2014) | 365 | Nutrient concentrations: Hb (g/dL), MCV (fL), MMA (μmol/L), plasma vit B12 (nmol/L), Hcy (μmol/L), Erythrocyte folate (nmol/L) | Multivariable log binomial regression | Deficiency cut-offs: • Anemia: Hb <11.0 • Severe anemia: Hb <7.0 • Microcytic anemia: anemia+MCV <80 • MMA levels >0.26 • Low plasma vit B12 <150 • Hcy levels >10.0 • Erythrocyte folate <283 | K-10; ≥6 vs. <6 | K-10 ≥6 vs. <6; chi-squared test: • Anemia, severe aemia, microcytic anemia significant, not shown • Macrocytosis: NS • Low vitamin B12 status: NS • Erythrocyte folate status: NS • Homocysteine status: NS • MMA status: NS K-10 ≥6 vs. <6; log binomial regression:  • Anemia: PR 0.66 (CI 0.46--0.95), p=0.024 | K-10 ≥6 vs. <6; log regression: • Anemia: PR 0.67 (CI 0.47--0.96), p=0.029 | Age, education*, occupation, parity, BMI, anemia*, nausea*, vomiting* | • History of depression not included • Protective association of anemia not discussed |
| Watanabe (2010) | 86 | Nutrient concentrations: Serum folate (ng/mL), plasma Hcy (nmol/mL), total protein (g/dL), Albumin (g/dL), serum iron (μg/dL), Hb (g/dL), Hct (%) | Linear correlation (all biomarkers), univariable logistic regression (only folate and Hcy) | Continuous, folate and Hcy also dichotomized at median: • Folate <8.1 vs. ≥8.1 • Hcy <6.1 vs. ≥6.1 | CES-D ≥ 16 vs. <16 | Correlation of nutrient and EPDS score: Total protein: r= 0.263, p=0.014 • Hb: r= 0.222, p=0.04 • Folate, Hcy, Albumin, Iron, Hct: NS CES-D score ≥16 vs.<16 logistic regression:  • Folate NS • Hcy NS | None significant | Age, height, pre-pregnancy weight, pre-pregnancy BMI, gestational age*, parity*, vomits/d*. | • Unrealistically high depression prevalence  • Prevalence of nutrient deficiencies not reported • History of depression not included • Depression measured in 1st trimester |
| • Depression terms: BDI: Beck's Depression Inventory; CES-D = Center for Epidemiological Studies Depression Scale; EPDS = Edinburgh Postpartum Depression Scale; K-10: Kessler Depression Scale; MDD: Major Depressive Disorder; PPD: Postpartum depression • Measurement terms: avg: Average; d: day(s); m: month(s); tri: trimester(s); w: week(s); y: year(s); g: grams; mg/l: milligrams per liter; μg/l: micrograms per liter; μmol: millimole; nmol: nanomole  • Statistical terms: *Significant (p < 0.05) in final model; CI: confidence interval; NR: Not reported; NS: not significant; OR: odds ratio; PR: prevalence ratio; Ref: Reference; RCT = randomized control trial  • Nutrient terms: A: retinol; AA: arachidonic acid; BMI: Body Mass Index; C: ascorbic acid; D: serum 25-hydroxyvitamin D; DHA: Docosahexaenoic acid; EPA: Eicosapentaenoic acid; EFA: Essential Fatty Acids; Hcy: homocysteine; Hct: Hematocrit; MMA: methylmalonic acid; MCV: mean corpuscular volume; sTfR: soluble transferrin receptors; Vit: Vitamin/s | | | | | | | | | |

**Reference from Supplementary Appendix S1**

1. Bennett HA, Einarson A, Taddio A, Koren G, Einarson TR. Prevalence of depression during pregnancy: systematic review. Obstet Gynecol. 2004;103(4):698-709.
